# Functional classification and mutation analysis of a synpolydactyly kindred

**DOI:** 10.3892/etm.2014.1957

**Published:** 2014-09-11

**Authors:** JIANDA ZHOU, YAO CHEN, KE CAO, YONGHUA ZOU, HAIYAN ZHOU, FENG HU, BIN NI, YONG CHEN

**Affiliations:** 1 Department of Plastic Surgery, Third Xiangya Hospital, Central South University, Changsha, Hunan 410013, P.R. China; 2 Key Laboratory of Genetics and Birth Health of Hunan Province, Family Planning Institute of Hunan Province, Changsha, Hunan 410078, P.R. China

**Keywords:** syndactyly, polydactyly, *HOXD13* gene, linkage, functional classification

## Abstract

The aim of the present study was to analyze a congenital syndactyly/polydactyly kindred and propose a new functional classification method of clinical significance. The modes of inheritance and mutational mechanisms were also determined using genetic analyses. Hand and foot anatomy and functions were measured using photographic images, X-ray imaging and grip ability tests. Genetic analysis comprised the genotyping of polymorphic microsatellite markers at known polydactyly-associated loci and the sequencing of the candidate gene. A functional classification system was devised to divide the clinical features into three types, which included mild, moderate or severe deformity. The family was concluded to have syndactyly type II with autosomal dominant inheritance. The microsatellites, D2S2310 and D2S2314, at the 2q31–32 chromosome, which have previously been associated with synpolydactyly type I, were found to be associated with the disorder in the current family. A 27-bp insertion mutation was identified in the affected individuals in the *HOXD13* gene at this locus. The insertion added a further nine alanine residues to the polyalanine stretch within the encoded protein. In conclusion, the functional classification method described in the present study may be used to guide surgical approaches to treatment. A family was identified in whom expansion of the polyalanine tract in the *HOXD13* gene causes autosomal dominant hereditary synpolydactyly.

## Introduction

Syndactyly/polydactyly, a congenital anomaly of the hands and feet, is one of the most common types of limb deformity, and is characterized by fusion of the bone or soft tissue between adjacent fingers or toes. Based on the differences in the fingers or toes involved, non-syndromic syndactyly/polydactyly is classified into five types according to Temtamy and McKusick’s method (1). Syndactyly/polydactyly type II is termed synpolydactyly with autosomal dominant inheritance.

Synpolydactyly is the joint presentation of syndactyly (fusion of fingers or toes) and polydactyly (supplementary digits), characterized by incomplete penetrance and inconsistent expressivity. In the current study, a synpolydactyly kindred from Huaihua in China was examined. Based on the clinical features and the degree of difficulty in deformity repair, a simple and feasible method for functional classification was proposed. Genetic linkage analysis was also performed at polymorphic microsatellite loci and a candidate gene was sequenced in the implicated chromosomal region.

## Materials and methods

### Family survey, functional classification and specimen collection

Informed consent was obtained from all family members prior to participation in the study. The kindred with congenital synpolydactyly was from Huaihua (Hunan, China) and consisted of three generations, totaling 39 members, including 19 affected members (4 males and 15 females) and 20 unaffected members. The 19 affected members were medically examined, and images and X-rays were obtained of their hands and feet. Furthermore, a 5-ml peripheral blood sample was conventionally extracted and placed in a vacuum heparinized tube. The present study was approved by the ethics committee of the Family Planning Institute of Hunan Province (Changsha, China).

As there were no marked foot dysfunctions involving walking, standing or running, only the hand deformities were grouped into mild, moderate or severe deformity categories, according to the functional classification method established in the present study. A mild deformity was classified as the possible presence of supplementary fingers, a normal outline of the fingers and joints, and no bone abnormalities or dysfunctions. A moderate deformity was classified as the possible presence of supplementary fingers and a normal shape and position of the metacarpals and phalanges. There were no bone adhesions and the grip strength was normal; however, webbed fingers or finger adhesions, as well as abnormal joint flexion, extension or other joint abnormalities, may have been present. A severe deformity was classified as the possible presence of supplementary fingers, with marked deformities or joint abnormalities, including metacarpal or phalangeal adhesions, and an abnormal grip function.

### Linkage analysis

#### DNA extraction and determination

A standard phenol-chloroform method was used to extract the total genomic DNA, which was quantified using a UV spectrophotometer (DU800, Beckman Coulter, Kraemer Boulevard Brea, CA, USA), and subsequently stored at −20°C.

#### Selection of polymorphic microsatellite DNA loci

Polymorphic microsatellite markers were selected from the three identified loci for synpolydactyly at chromosomes 2q31, 22q13.31 and 14q11.2–q12. The markers and their corresponding fluorescence-labeled primers (Sunny Biotech Co., Ltd., Shanghai, China) are shown in Table I.

#### Polymerase chain reaction (PCR) amplification of the microsatellite sequences and genotyping

The microsatellites were amplified by multiplex PCR. According to the length of the products, an appropriate intramolecular marker (0.12 μl; GeneScan^™^ 500 TAMRA^™^; Applied Biosystems^®^; Invitrogen Life Technologies, Foster City, CA, USA) was selected and mixed with high-purity carboxamide (4 μl) and the PCR product (1 μl). Following 3 min denaturation at 95°C, the mixture was cooled on ice to maintain the single-stranded DNA. The mixture was subjected to capillary electrophoresis on an ABI Prism^®^ 310 Genetic analyzer (Applied Biosystems^®^) for 2 h.

#### Data analysis

Data were collected using GeneScan 2.1 software (Applied Biosystems^®^) and subjected to lane line correction. The collected gel images were converted to digital signals for internal calibration of the molecular weight and analysis of the amplified fragment sizes.

#### HOXD13 gene mutation analysis

A proband and an unaffected relative were selected from the family for mutation detection in the *HOXD13* gene. The online software, Primer3, was used to design primers to amplify the exons and intron/exon boundaries of the gene (Table II). The PCR products obtained were purified by agarose gel electrophoresis, quantified and sequenced using the two primers. The obtained sequences were compared with the reference gene sequence in GenBank (accession no. NM_000523). The identified mutation was verified experimentally in all family members.

## Results

### Genetic analysis and clinical phenotype of the pedigree

**Figure 1:**
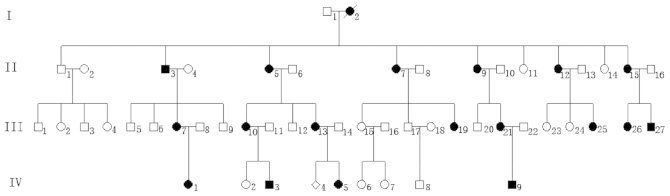
Pedigree of a Chinese kindred with congenital synpolydactyly. The kindred comprises three generations, totaling 39 members, including 19 affected members (4 males and 15 females).

**Figure 2:**
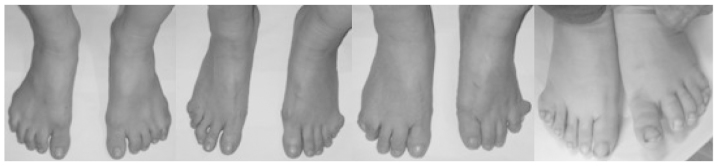
Clinical symptoms in the patients with toe deformities. There was no significant dysfunction of the toes in terms of standing, walking or running.

**Figure 3:**
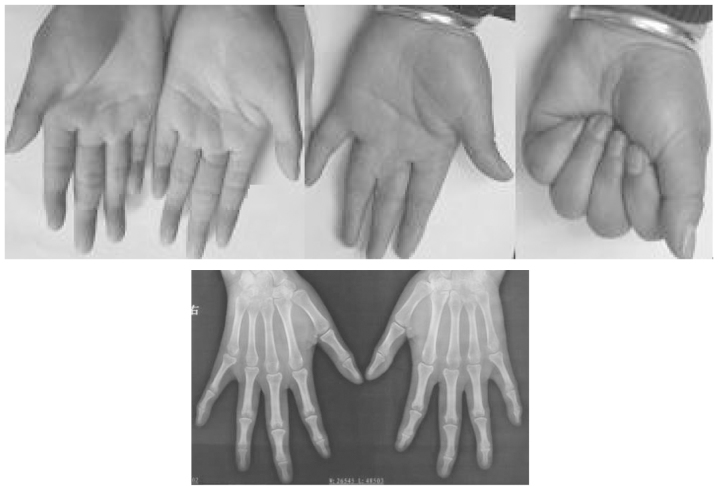
Images of mild deformities. There may or may not be supplementary fingers, the outline of the fingers and joints is normal and there are no bone abnormalities or dysfunctions.

**Figure 4:**
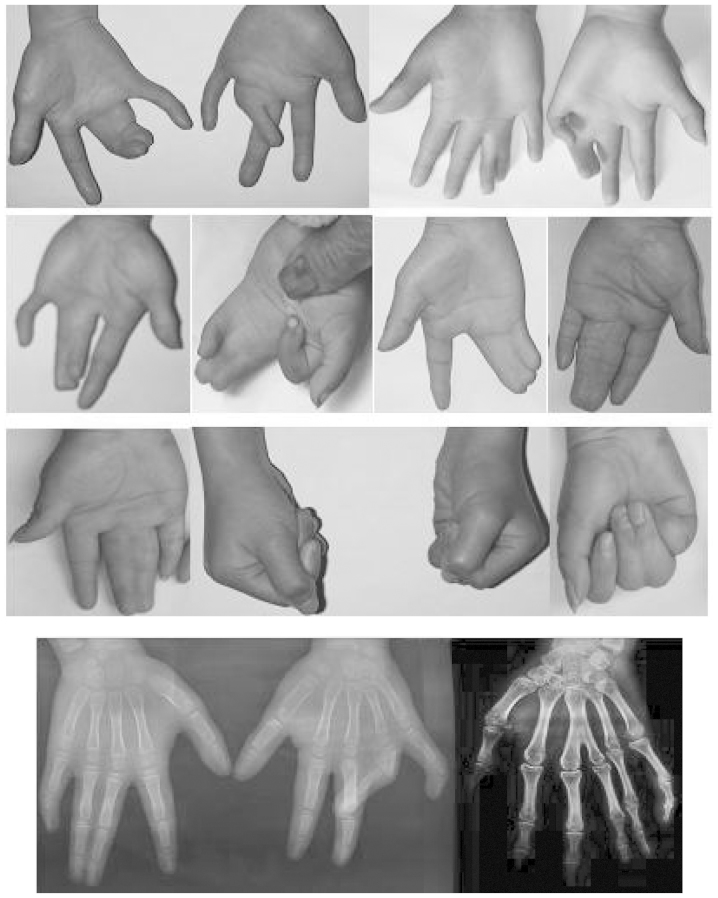
Images of moderate deformities. There may or may not be supplementary fingers, the shape and position of the metacarpals and phalanges are normal, there are no bone adhesions and grip strength is normal. However, there may be webbed fingers or finger adhesions, as well as abnormal joint flexion, extension or other joint abnormalities.

**Figure 5:**
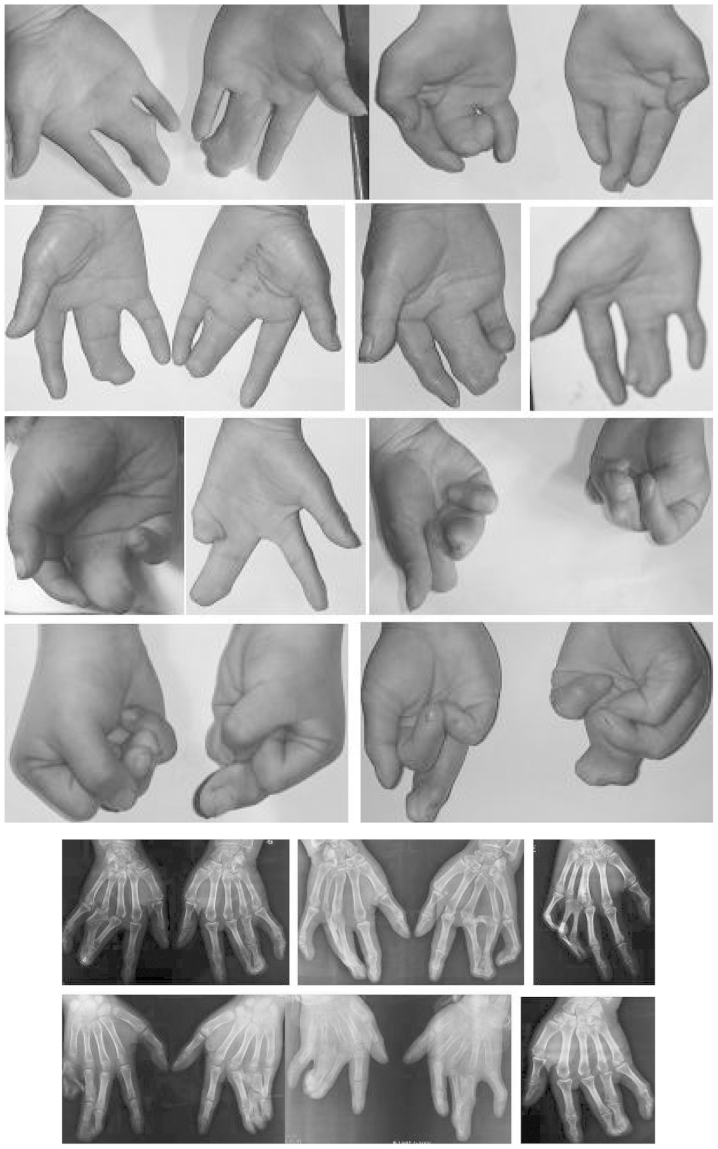
Images of severe deformities. There may or may not be supplementary fingers, there are evident deformities or joint abnormalities, including metacarpal or phalangeal adhesions, and the grip function is abnormal.

The clinical phenotypes of the kindred were characterized as follows: i) Disease inheritance within the kindred; ii) one parent of the proband is also affected; iii) offspring of unaffected individuals are always unaffected; and iv) equal opportunity for male and female members to be affected. The disease was shown to be inherited in an autosomal dominant manner (Fig. 1). The patients revealed no deformities other than those of the hands and feet; however, the phenotypes of the affected members were complex, with variation in the expressivity. There were significant phenotypic differences between the hands and feet of the affected members. No significant dysfunction was observed in the toes that affected standing, walking or running (Fig. 2). In the 38 hands of the 19 affected patients, the number of hands with a mild (Fig. 3), moderate (Fig. 4) and severe (Fig. 5) deformity were 3, 17 and 18, respectively. This demonstrates that the clinical features of the feet and hands have significant differences in a pedigree with individuals of an identical genotype.

### Family linkage analysis

D2S2310 and D2S2314, genetic markers closely associated with synpolydactyly type I, were genotyped in all the affected members. D14S264, D14S283, D22S444 and D22S1170 revealed a non-genetic linkage.

### Mutation analysis of HOXD13

Sequencing of the *HOXD13* PCR products revealed that all the affected family members had a 27-bp insertion encoding nine additional alanine residues. No other mutation was identified in the coding region.

## Discussion

Non-syndromic polydactyly is usually inherited in an autosomal dominant manner; however, the condition has been reported to demonstrate autosomal recessive inheritance in certain studies (2). Currently, there are a number of classification methods that are based on anatomical classification (3). These include Temtamy and McKusick’s method, which mainly emphasizes the relevance between genotyping and the clinical phenotype of syndactyly/polydactyly. This method has been accepted and utilized by subsequent practitioners. However, this method has limitations when selecting the clinical surgical method due to a complicated and meticulous classification process that requires the promotion of mutual communication between clinical staff. Therefore, functional classification and sorting according to surgical difficulty are recommended. In the current study, a method of clinical classification, regardless of gene variation type, was established. The classification was based only on clinical phenotype and the severity of the syndactyly/polydactyly; thus, may guide the selection of the clinical surgery method.

As there were no evident foot dysfunctions involving walking, standing or running, the hand deformities only were functionally grouped into mild, moderate or severe deformity categories. Patients with a joint deformity and bone abnormalities in the hands exhibited marked dysfunction of the hands. For a mild deformity, finger resection and corrective surgery are usually sufficient for treatment. For a moderate deformity, syndactyly separation, webbing enlargement and articular repair are recommended. Surgical treatment for a severe deformity is complex, including osteotomy, bone separation, skin grafting, flap surgery and joint reconstruction. The type of classification method used in the present study may also have its application extended to all cases of syndactyly/polydactyly.

**Figure 6:**
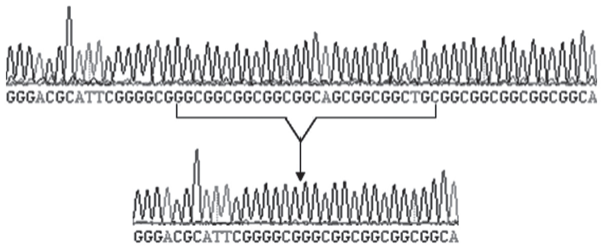
Sequencing results of the *HOXD13* gene from affected and unaffected individuals. Compared with the reference *HOXD13* gene sequence (GenBank accession no. NM_000523), the affected members all carried a 27-bp insertion, encoding nine additional alanines.

Synpolydactyly types I, II, and III have been mapped to the 2q31, 22q13.31 and 14q11.2–q12 chromosomes, respectively (4–6). HOX genes encode a highly conserved family of transcription factors that control the formation of the primary and secondary body axes during embryonic development (7). The 39 Hox genes are generally arranged into four clusters: HOXA, HOXB, HOXC and HOXD, which are located at chromosomes 7p14, 17q21, 12q13 and 2q31, respectively (8). Each gene cluster is ~120 kb and contains 9–11 genes (9–10). Mutations in the *HOXD13* gene have been previously associated with synpolydactyly (11–13). All HOX proteins are capable of binding to specific DNA sequences via a DNA-binding motif consisting of 60 amino acids. *HOXD13* comprises two exons with a coding region of 1,008 bp, and encodes a protein of 335 amino acids (14), containing a homeobox at the 3′ end and two polyserine chains and one polyalanine chain at the 5′ end. The polyalanine chain, which usually has a length of 15 residues, has been found to be expanded in cases of synpolydactyly (7,15–19), possibly resulting from the unequal crossover of imperfect trinucleotide repeats in the GCN codon and exon 1 of *HOXD13* (20,21). An abnormally long polyalanine chain results in the migration of the HOXD13 protein from the nucleus to the cytoplasm, which is followed by abnormal protein aggregation and altered transcription factor activity (23). Goodman *et al* (22) observed that the penetrance in 20 typical synpolydactyly pedigrees was positively associated with the severity of limb deformities and the number of alanine residues. Other *HOXD13* mutation types have also been associated with synpolydactyly, including nonsense (24,25) and missense (12) mutations, small deletions, frameshift mutations and splice acceptor mutations (17,26,27). From the genotyping results of the microsatellites in these regions, the causative gene in the current pedigree was mapped to 2q31 (type I). The first identified repetitive sequence of c.168_194dup was GGCGGCGGCGGCGGCAGCGGCGGCTGC (27 bp; Fig 6); however, its encoded amino acid sequence was the same as that reported by Goodman *et al* (22).

In conclusion, the cause of synpolydactyly in the Chinese family examined in the present study was identified to be a polyalanine expansion in the *HOXD13* gene. Penetrance in the family was up to 100%; however, expressivity was inconsistent between individuals and even between the hands and feet of the same individual, with certain patients exhibiting different severity of synpolydactyly between different phalangeal joints. The results of the current study may be useful for prenatal genetic and molecular diagnosis of synpolydactyly.

## Figures and Tables

**Table I:** Primer sequences, fragment size and chromosomal localization.

Genetic marker	Primer sequences	Fragment size (bp)	Chromosomal localization
D2S2314	5′ FAM-GGTGTCAGTGAGACCCTGT 3′	96–118	
	5′ ATTTCTAGCGGCCCTAAAAC 3′		
D2S2310	5′ FAM-CGACTTGAGTAGACGCACTATTC 3′	244–260	
	5′ GCATCTAAACTGTGAAATGAGC 3′		2q31–32
D22S444	5′ FAM-TTTGAACTAAGCCTTAAAAATGC 3′	123–131	
	5′ TGTTTGGCTTGAAGAAGGAG 3′		
D22S1170	5′ FAM-ACCGTTGCCTATATCCA 3′	180–212	
	5′ AGCCCACTCCACAATTT 3′		22q13–14
D14S264	5′ FAM-CCCCAAATATCACTCCAAAT 3′	216–234	
	5′ GAGTTGGCAACCACTTCTGT 3′		
D14S283	5′ FAM-GGGACTATATCTCCCAGGC 3′	125–153	
	5′ TGTTTTCCTAGTAACCGCA 3′		14q11–12

**Table II:** Primers for *HOXD13* mutation analysis and polymerase chain reaction fragment sizes.

Primer	Upstream	Downstream	Fragment size (bp)
Hoxd13-1	5′ GAGAAAGGAGAGGAGGGAGGAG 3′	5′ AGGGCTCGTATAGCCCTGGT 3′	684
Hoxd13-2	5′ GGCTCTAAATCAGCCGGACA 3′	5′ GGCAACTGCTGAGAGCTAATGA 3′	727
Hoxd13-3	5′ CCGGCTATATCGACATGGTGT 3′	5′ CATGTCCGGCTGATTTAGAGC 3′	1008
